# The role of the testa during development and in establishment of dormancy of the legume seed

**DOI:** 10.3389/fpls.2014.00351

**Published:** 2014-07-17

**Authors:** Petr Smýkal, Vanessa Vernoud, Matthew W. Blair, Aleš Soukup, Richard D. Thompson

**Affiliations:** ^1^Department of Botany, Faculty of Sciences, Palacký University in OlomoucOlomouc, Czech Republic; ^2^INRA, UMR 1347 AgroécologieDijon, France; ^3^Department of Agricultural and Environmental Sciences, Tennessee State UniversityNashville, TN, USA; ^4^Department of Experimental Plant Biology, Charles UniversityPrague, Czech Republic

**Keywords:** domestication, dormancy, hardseededness, legumes, proanthocyanidins, seed coat, testa, water permeability

## Abstract

Timing of seed germination is one of the key steps in plant life cycles. It determines the beginning of plant growth in natural or agricultural ecosystems. In the wild, many seeds exhibit dormancy and will only germinate after exposure to certain environmental conditions. In contrast, crop seeds germinate as soon as they are imbibed usually at planting time. These domestication-triggered changes represent adaptations to cultivation and human harvesting. Germination is one of the common sets of traits recorded in different crops and termed the “domestication syndrome.” Moreover, legume seed imbibition has a crucial role in cooking properties. Different seed dormancy classes exist among plant species. Physical dormancy (often called hardseededness), as found in legumes, involves the development of a water-impermeable seed coat, caused by the presence of phenolics- and suberin-impregnated layers of palisade cells. The dormancy release mechanism primarily involves seed responses to temperature changes in the habitat, resulting in testa permeability to water. The underlying genetic controls in legumes have not been identified yet. However, positive correlation was shown between phenolics content (e.g., pigmentation), the requirement for oxidation and the activity of catechol oxidase in relation to pea seed dormancy, while epicatechin levels showed a significant positive correlation with soybean hardseededness. myeloblastosis family of transcription factors, WD40 proteins and enzymes of the anthocyanin biosynthesis pathway were involved in seed testa color in soybean, pea and *Medicago*, but were not tested directly in relation to seed dormancy. These phenolic compounds play important roles in defense against pathogens, as well as affecting the nutritional quality of products, and because of their health benefits, they are of industrial and medicinal interest. In this review, we discuss the role of the testa in mediating legume seed germination, with a focus on structural and chemical aspects.

## LEGUMES

Legume seeds are the second most important plant protein source, on a world basis, after cereals. While in cereals the major storage molecule is starch, which is deposited in the endosperm, in most of the grain legumes (pulses) the endosperm is transitory and consumed by the embryo during seed maturation, which contains a high proportion of proteins (20–40%), and either lipids (soybean, peanut) or starch (or both) as a further carbon source. Nutritionally, they are generally deficient in sulfur-containing amino acids (cysteine and methionine), but unlike cereal grains, their lysine content is relatively high. The major storage proteins are globulins, which account for up to 70% of the total seed nitrogen. The ability of legumes to fix atmospheric nitrogen allows them to colonize poor soils; however adequate nitrogen reserves in the seed are vital to allow the seedling to survive the heterotrophic growth phase before nitrogen fixation is established in root nodules. Fabaceae, the third largest family of flowering plants, are divided into three subfamilies: Caesalpinioideae, Mimosoideae, and Papilionoideae, all together with 800 genera and 20,000 species (Lewis et al., 2005). The latter subfamily contains most of the cultivated major food and feed crops (Smýkal et al., 2014). It is a diverse family with a worldwide distribution, encompassing a broad range of plant forms, from annual and perennial herbs to trees and lianas. This variation is also reflected by widely diverse seed shapes and sizes, ranging from 1-mm seeds of the native Australian legume species *Pycnospora lutescens* to 18-cm seeds of the coastal tree *Mora oleifera* (Gunn, 1981). Legume seeds develop either within pods (e.g., legume), or less frequently, samara (Lewis et al., 2005), which can have several functions; protective, dispersal, and nutritive, can comprise a significant source of remobilized nutrients during seed filling.

## DORMANCY CONCEPTS

The function of a seed is to establish a new plant but it can do this only once, because the completion of germination essentially is an irreversible process. Plants have evolved several dormancy mechanisms to optimize the time of germination (Foley, 2001). Since seed dormancy is a physiological adaptation to environmental heterogeneity, it is a primary factor that influences natural population dynamics (reviewed in Bewley et al., 2013). Dormancy provides a strategy for seeds to spread germination in time in order to reduce the risk of plant death and possible species extinction in an unfavorable environment. Dormancy occurs in three ways: (1) Seeds are dispersed from the parent plant with different degrees of dormancy. Frequently, the variation in dormancy is reflected by the appearance of the seeds or dispersal units in terms of color, size, and thickness of the coat. (2) Through the dependence of dormancy breakage on environmental factors. (3) Through seed dispersal via animals, wind or water (reviewed in Bewley et al., 2013). A classical concept of seed dormancy was formulated by Harper (1957) who distinguished three types: (1) Seeds born as dormant (innate); (2) Those with achieved dormancy (induced); and (3) Seeds with dormancy thrust upon them (enforced). Moreover, Harper distinguished two categories of plants living in a community; those which are growing at present and those which are dormant (in the form of a soil seed bank). A problem in distinguishing dormancy-relieving factors from factors stimulating or initiating germination is that the actual state of dormancy cannot be measured directly (Thompson and Ooi, 2013). Vleeshouwers et al. (1995) defined dormancy as: “a seed characteristic, the degree of which defines what conditions should be met to make the seed germinate” (Vleeshouwers et al., 1995). Consequently, seeds of many species that form a persistent seed bank exhibit annual changes in dormancy (Karssen, 1982; Milberg and Andersson, 1997). This phenomenon of dormancy cycling (Baskin and Baskin, 1985) is regulated by various factors such as temperature. Moreover, the intensity of dormancy within a given species varies at several levels: among populations, within populations and between seeds collected in different years from the same population (Foley, 2001; Lacerda et al., 2004). There is also heterogeneity in dormancy among seeds at the level of the individual plant (reviewed in Matilla et al., 2005), depending on the age and the nutritional status of the mother plant during seed maturation, seed position on the mother plant, seed size and shape, the time since seed harvest, and the duration of seed storage (Probert, 2000). Despite of all this variation, seed dormancy has a clear genetic basis (Graeber et al., 2012). Several dormancy classes were defined by Nikolaeva (1969, 1977) and more recently reviewed by Finch-Savage and Leubner-Metzger (2006). Morphological dormancy refers to seeds that have an underdeveloped embryo and require longer time to grow and germinate. Physiological dormancy, the most prevalent form of dormancy, appears to broadly involve abscisic acid (ABA) and gibberellins (GAs) metabolism. In addition, there are morphophysiological and combinational dormancies. In contrast to hormone-mediated seed dormancy, extensively studied in *Arabidopsis* or cereals, we have still limited knowledge of the regulation of physical dormancy, which involves the development of a water-impermeable seed coat (Baskin et al., 2000). This type of dormancy is found in at least 17 plant families, including agronomically important families like the Fabaceae, Malvaceae, Cannaceae, Geraniaceae, and Convolvulaceae (Baskin et al., 2000) and is present in the wild progenitors of cultivated legumes (Dueberrn de Sousa and Marcos-Filho, 2001; Zohary et al., 2012; Abbo et al., 2014).

Germination begins with water uptake (imbibition) by the quiescent dry seed and is completed by radicle protrusion through the tissues surrounding the embryo. There are three phases of seed imbibition. Dry seeds have very low water potentials, which causes rapid water influx during phase I. As this process is driven by water potential, it also occurs in dead seeds. Similar phenomena can be observed in resurrection plants and pollen. The permeability of the testa, being the part of the seed that comes into contact with the ambient water, plays a central role in water uptake. Phase II encompasses the rupture of the testa, and during phase III endosperm rupture and radicle protrusion occur (Finch-Savage and Leubner-Metzger, 2006). Germination occurs when embryo growth overcomes the constraints imposed by the seed coat (Bewley et al., 2013).

In nature, exposure to high temperature or fluctuating temperatures is the most likely cause of release from seed dormancy. The interactions between seed dormancy mechanisms and accumulated and current environmental conditions determine whether and what fraction of seeds in a seed bank will germinate at a given time. Weather and soil physical characteristics largely determine the microclimate to which seeds are exposed. The most critical environmental factor is water. Seeds imbibe water from their surroundings, and the water potential of the soil determines the maximum water potential that the seeds can attain. Seed banks may be composed of seeds from different years, which experienced different after ripening- or dormancy-breaking regimes, resulting in multiple subpopulations with different dormancy characteristics. Temperature is the second most important environmental determinant of seed germination. In extreme cases this is associated with fire (Hanley and Fenner, 1998) or vegetation gaps that are hotter than the surrounding forest soils (Vázquez-Yanes and Orozco-Segovia, 1982). Moreover, water and temperature regimes interact and also light plays a role in the onset of germination via regulation of phytochrome activity. Other factors include oxygen and other gasses. Dependence on exogenous factors for the initiation of germination suggests that physically dormant seeds should be limited in their ability to spread germination risk over multiple time periods or “recruitment opportunities” (reviewed in Bewley et al., 2013). However, in a few taxa the responsiveness of seeds to dormancy-breaking cues varies seasonally (Karssen, 1982), which suggests temperature, rainfall and perhaps deciduous tree leaf-drop as key factors. In summary, seasonal germination patterns are largely controlled by the seeds’ responses to prevailing environmental factors, such as moisture, temperature, light, and various chemicals in conjunction with seasonal environmental factors (e.g., chilling, after-ripening) that sensitize the seeds to the environment.

## LEGUME SEED COAT (TESTA) DEVELOPMENT AND STRUCTURE

The angiosperm seed develops from the fertilized ovule and depending on the stage of development is usually composed of (1) the embryo, arising by fertilization of the egg cell by one of the pollen tube nuclei; (2) the nutritive tissue of the endosperm, generated by the fusion of two polar nuclei of the embryo sac with the other sperm nucleus; and (3) a protective seed coat (testa), derived from the inner, outer or both ovular integuments (Bradford and Nonogaki, 2009).

### THE SEED COAT TISSUE COMPONENTS

**FIGURE 1:**
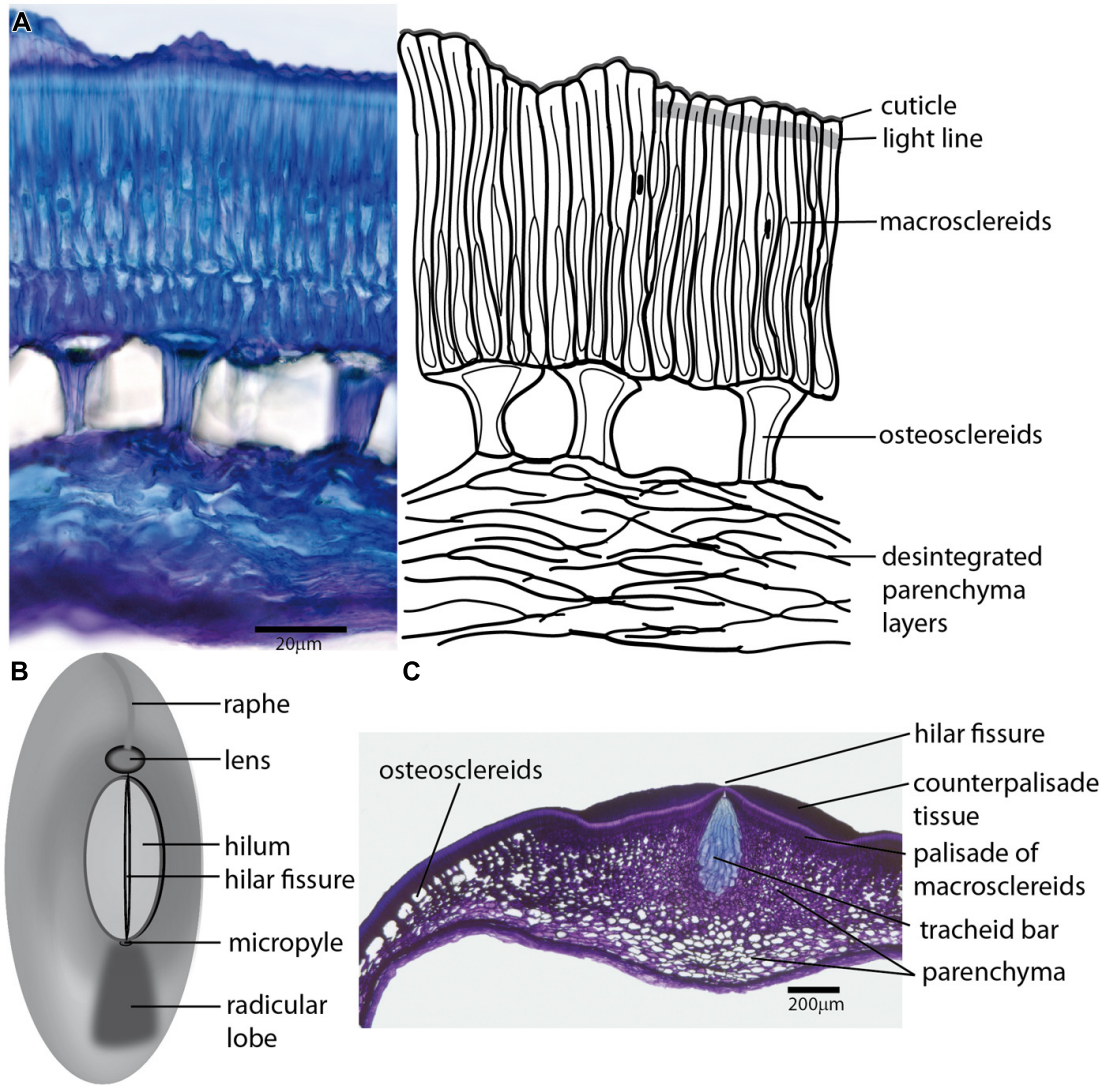
**The arrangement of Fabaceae seed coat share rather common structural features. (A)** Transversal section of the seed coat of wild *Pisum sativum* subsp. *elatius* (left), with a schematic drawing (right); epidermal cells differentiate into macrosclereids, which are characterized by a cuticle-covered surface. The outer parts of the macrosclereids (sclereid caps) are frequently separated by a region of the cell wall (light line) with specific features, resulting in different optical and staining properties. The central part of the testa differentiates into osteosclereids with a specific shape caused by thickened secondary cell wall. The innermost layers of parechymatous cells frequently die during differentiation and only disintegrated fragments are left. **(B)** Generalized scheme of the seed coat morphology commonly found in Fabaceae seeds showing the most important structural features, including the hilum, lens differentiated on the raphe and micropylar pore. **(C)** Transversal section of a *Pisum sativum* seed coat in the area of the hilum. The macrosclereids of the hilar scar are covered with counter palisade tissue with a central fissure above the tracheid bar, which is surrounded by star-shaped parenchyma interconnected to intercellular spaces of a layer of osteosclereids.

The origin of the seed coat can be traced back to the L1 sporophyte layer of the ovule primordium (Schneitz et al., 1997) with an emerging network of regulatory pathways coordinating growth of the inner and/or outer integuments surrounding the ovule (Skinner et al., 2004; Galbiati et al., 2013; Kurdyukov et al., 2014). The number of ovule integuments varies depending on the species; legumes have two integuments (bitegmic ovules). The inner integument largely vanishes during development (Esau, 1965) while the outer one produces several distinct cell layers and establishes the “typical” seed coat structure. The chalazal region is an important part of the testa where connections of the vascular tissues of the maternal funiculus terminate. The scar where the funiculus was attached is called hilum (**Figure 1B**). Current seed identification criteria are based upon morphological characteristics including seed size, general shape, surface shape, color, pattern, hilum length and width. These are often used in taxonomical classifications (Lestern and Gunn, 1981; Chernoff et al., 1992; Güneş, 2013) and archeobotany (Zohary et al., 2012). Legume seed characters support the concept of one family (Fabaceae) as advocated already by de Candolle (1825). Although the seed coats of different species vary greatly in structure and composition, they undergo similar phases of development in relation to the embryo and endosperm (Butler, 1988). In legumes, the seed coat and endosperm develop first, followed by development of the embryo (Weber et al., 2005).

In spite of some known exceptions such as peanut (*Arachis hypogaea*) with lignified pod or *Archidendron* and *Pithecellobium*, which have a partly pulpy and edible testa (Gunn, 1981), there is a rather common blueprint of seed coat structure for the Fabaceae family (Lush and Evans, 1980). Interspecific variation comprises mainly the patterns of differentiation, dimensions, and modifications of cell walls of individual layers. There are a number of publications in this area (Pammel, 1899; Rolston, 1978; Lush and Evans, 1980) with considerable emphasis on economically important soybean.

### THE OUTER INTEGUMENTS

The epidermis of the outer integument forms a single layer of tightly packed palisade of radially elongated sclereids (called Malpighian cells, macrosclereids, or palisade cells) with heavily and unevenly thickened cell walls (**Figure 1A**). The outer tangential cell walls are covered with cuticle and, because of specific cell wall thickening and modifications, they are commonly described as terminal caps. Their shape together with cuticle and waxy depositions determine the texture of the seed coat surface (Güneş, 2013). The architecture of this layer and the structure of its cuticle attract considerable attention as their properties are generally related to water-impermeability of hard seeds (White, 1908; Hamly, 1932, 1935; Riggio Bevilacqua et al., 1989; Argel and Paton, 1999). The cuticle forms a continuous layer covering the seed, except for the hilum, and is considered the outermost barrier to imbibition (White, 1908; Spurny, 1963; Ma et al., 2004; Shao et al., 2007). Interestingly, the chemical composition of soybean cuticle seems to differ in fatty acid composition from the shoot cuticle (Shao et al., 2007). Its continuity, being a crucial feature of hardseededness, might be compromised by the emergence of cracks during seed expansion and development (Ranathunge et al., 2010). In some legume species, such as soybean, the seed surface is also modified by secretory activity of the fruit (pod) wall (Yaklich et al., 1986) which might deposit hydrophobic proteins on the cuticle (Clarkson and Robards, 1975; Gijzen et al., 1999).

### THE SCLEREID LAYERS

There is frequently a lucent region of macrosclereid cell walls separating macrosclereid terminal caps from their basal parts. This border line, which extends transversally across the macrosclereid layer is named light-line or *linea lucida* (**Figure 1A**) in many species. Its appearance derives from local variation in refractive indices and stainability attributed to the modifications in polysaccharide deposition and/or impregnation of this cell wall region (Hamly, 1935; Harris, 1983; Bhalla and Slattery, 1984; Bevilacqua et al., 1987). The strength of the light line was related to seed coat impermeability (Stevenson, 1937; Harris, 1987). The lumen of macrosclereids is usually irregular and tapered toward the seed coat surface due to prominent cell wall thickening. Macrosclereids differentiation was followed in cytological detail in pea (Spurny, 1963; Harris, 1983), soybean (Harris, 1987), and clover (Algan and Büyükkartal, 2000). The length of macrosclereids seems to be under environmental control in soybean (Noodén et al., 1985). Unevenly distributed pores (pits) in soybean seed coat develop during the desiccation phase (Yaklich et al., 1986; Vaughan et al., 1987) and the presence of such cracks, pits and other irregularities on the seed coat surface, seems to be related to its water permeability (Wolf et al., 1981; Yaklich et al., 1986; Ma et al., 2004). A subepidermal layer of cells is differentiated into osteosclereids (bone-shaped cells), also termed by different authors columnar or pillar cells, hourglass cells or lagenosclereids (flask-shaped cells) depending on the shape of the cells. This layer includes conspicuous air-filled intercellular spaces (**Figure 1A**) resulting from cell shaping during testa differentiation and massive cell wall deposition in the middle part (Harris, 1983; Miller et al., 2010). Osteosclereids are the first major cell types during testa differentiation where cell death was detected, followed by parenchyma and macrosclereids (Ranathunge et al., 2010). In and around the hilum, the layer of osteosclereids merges with thick-walled, star-shaped parenchyma (**Figure 1C**). Continuity of the intercellular spaces may be related to seed desiccation and gas exchange.

### THE PARENCHYMA OR NUTRIENT LAYER

The innermost part of the seed coat is composed of parenchyma cells (**Figure 1A**), which are elongated in tangential direction and result in abundant air-filled intercellular spaces. Frequently there are 5–12 cell layers of parenchyma with the inner layer in direct contact with the endosperm (Hamly, 1932, 1935). Some authors call the parenchymatous region “nutrient layer” owing to its function during embryo development (Van Dongen et al., 2003). The seed coat vascular systems are embedded into parenchyma layers and their structures vary amongst legumes; some species possess an extensive vascular systems that anastomose to form reticulated networks throughout the entire seed coat (e.g., common bean, Oﬄer and Patrick, 1984; soybean, Thorne, 1981), while other species have relatively simple vascular systems, with only a single chalazal vascular bundle and two lateral branches extending into the seed coats (e.g., pea, Spurny, 1963; Hardham, 1976; broad bean, Oﬄer et al., 1989). During seed coat maturation, parenchyma cells lose the protoplast and the innermost layers might be crushed. Parenchyma layers are not generally related to water-impermeability. However, high callose levels in this layer might be related to low permeability of clover seed coat (Bhalla and Slattery, 1984), but a causal relationship remains to be determined.

### THE MICROPYLE AND HILUM

The anatomical structure of the testa is rather homogenous except for the chalazal region (**Figure 1B**). The hilum is a distinctly oval or round abscission scar in the chalazal seed region, a relic of former connection of the seed to the maternal plant via the funiculus. There is another residual layer of palisade cells of funicular origin termed counter-palisade, which are part of the hilar scar (Lackey, 1981). There is a central fissure (hilar groove) in the hilum palisade layer overlaying the tracheid bar (**Figures 1B,C**) from the micropyle to the ovular bundle on the other side. This strip of large, pitted and lignified tracheids is commonly found in legumes although there is some variability in structure and ovular bundle position (for more detail see Lersten, 1982). A role for the hilar groove as a hygroscopically activated valve was suggested (Hyde, 1954; Lush and Evans, 1980). The fissure in the hilum opens when relative humidity is low permitting the seed to dry out whereas high relative humidity causes the fissure to close preventing the absorption of moisture.

### THE LENS (STROPHIOLE)

The micropyle, an entrance pore for the pollen tube (**Figure 1B**), is inconspicuous on mimosoid and casealpinoid seeds but is discernible (often of different color) on faboid seeds (Gunn, 1981). In some species, there is a residue of the micropylar opening covered with a waxy lid (Vaughan et al., 1987). Specific outgrowth of raphe termed lens (strophiole) on the other side of the hilum (**Figure 1**) might be obvious in some species (Lersten and Gunn, 1982; Lersten et al., 1992). This structure is considered to act as a water gap (Baskin et al., 2000; Hu et al., 2009; Karaki et al., 2012) that might be open by external (e.g., heat, mechanical action, temperature variation; Baskin, 2003; Van Assche et al., 2003) or internal factors (Rolston, 1978; Argel and Paton, 1999). The palisade cells of the lens region are modified, narrower, longer and more variable than in the rest of testa. A loosely arranged cell structure on the lens-side of the hilum, a deeply grooved hilar fissure, and a narrow tracheid bar were considered to be associated with high initial water absorption in some common bean and *Psophocarpus* seeds (Deshpande and Cheryan, 1986).

Functionally, the main effects exerted by the tissues surrounding the embryo are: (1) the interference with water uptake, (2) mechanical restraint to radicle protrusion, (3) inference with gas exchange, (4) prevention of inhibitor leakage from the embryo, (5) supply of inhibitors to the embryo and 6) light penetration in species in which light plays a role in germination (Werker et al., 1979; Nowack et al., 2010; Bewley et al., 2013).

### COORDINATED GROWTH OF THE THREE SEED COMPONENTS

The coordinated growth of the inner and outer integuments, which ensures that the ovule is surrounded by protective tissues, is regulated by several locally expressed transcription factors (TFs), which were first identified in *Arabidopsis* (Skinner et al., 2004). The integuments are initially undifferentiated, but rapidly undergo changes to produce a complex structure that protects the embryo and sustains its growth. In fact, differentiation of the seed coat from the ovular integuments includes some of the most dramatic cellular changes observed during seed development. Early seed coat development from the time of anthesis to seed maturation has been well described in soybean (Miller et al., 1999), pea (Van Dongen et al., 2003), *Medicago truncatula* (Wang and Grusak, 2005), and faba bean (Oﬄer et al., 1989; Oﬄer and Patrick, 1993; Borisjuk et al., 1995), and shows a relative homogeneity in term of ontogeny and final structure. During early legume seed development, the embryo and endosperm develop within the seed coat. The endosperm and embryo divide in parallel, with the endosperm occupying a larger volume until the beginning of seed filling or maturation, when the endosperm begins to degenerate and the embryo cells expand to accumulate storage products. Importantly, early embryo development and differentiation are controlled by the surrounding maternal tissue, and signals from the maternal plant must be transmitted through the seed coat and endosperm before they can reach the embryo. A model for the maternal control of embryo development through sugar metabolism that implicated seed coat invertases has been developed in legumes (reviewed in Weber et al., 2005). In *Arabidopsis*, mutations of the endosperm-specific LRR kinase HAIKU or the WRKY transcription regulator MINISEED3 result in limited growth of the endosperm, which in turns affects cell elongation in the seed coat resulting in smaller seeds. Conversely, mutation of the maternally expressed TF TTG2 restricts cell elongation in the seed coat, which limits endosperm growth. Whether homologous genes play a role in legume seeds remains to be shown.

## DEVELOPMENT AND ACCUMULATION OF MAIN COMPONENTS OF THE TESTA

The different seed coat cell layers have three main physiological functions: (1) Production, transport, and download of metabolites for zygote development, including metabolite inter-conversions, transport of photosynthetic assimilates and photosynthesis, (2) synthesis and deposition of defense-related compounds, both phytoalexins and structural components, and (3) establishment of physical dormancy and mechanical protection. Seed coat development and structure in soybean and *Medicago truncatula* share some traits that differ from those of *Arabidopsis* (Miller et al., 1999; Wang and Grusak, 2005). Whereas *Arabidopsis* epidermal cells produce and secrete large quantities of mucilage, soybean and *Medicago* epidermal cells do not and show extensive cell wall thickening (Russi et al., 1992). The innermost seed coat cell layer, the endothelium, is a metabolically active cell layer and is the main site of synthesis of proanthocyanidins (PAs). In the *Arabidopsis* mature seed, a brown pigment layer (bpl) forms between the inner integument 1 (ii1) and the outer integument 1 (oi1) cell layers as a result of compaction of several parenchyma cell layers (Beeckman et al., 2000). The endothelium has been described in soybean (Miller et al., 2010) and was microdissected for transcriptomic analysis (Le et al., 2007). In faba bean, this layer has been termed thin-walled parenchyma (Miranda et al., 2001). Because the aleurone remains at maturity and is crushed against the innermost layer of the seed coat, it is often considered as part of the seed coat (Miller et al., 1999; Moïse et al., 2005). However, differences in their development and more importantly in their origin (maternal for the seed coat vs. zygotic for the aleurone) make these two tissues distinct entities with specific roles during seed development.

Seed coat development is tightly regulated, and several tissues present during embryogenesis and seed filling will not persist at maturity or will undergo important modifications. This is particularly true for the parenchyma layers derived from the inner ovular integuments, which proliferate and then collapse and are crushed (Miller et al., 1999; Nadeau et al., 2011). In pea, three parenchyma sub-layers can be distinguished during testa development: chlorenchyma, ground parenchyma, and branched parenchyma. Starch transiently accumulates in the plastids of these cell layers, the young seed coats being a transient storage organ for carbohydrates and proteins (Rochat and Boutin, 1992). A dramatic and transient expansion of the branched parenchyma occurs during the filling period followed by its complete compression and the formation of a boundary layer between the seed coat and the filial tissue (Van Dongen et al., 2003; Nadeau et al., 2011). In pea and common bean, branched parenchyma is the site of expression of extra-cellular invertases, and it has been suggested that the degradation of this layer initiates the storage phase through a switch from high to low ratios of hexose to sucrose in the developing seeds (Weber et al., 1997; Van Dongen et al., 2003). Important cellular changes are also observed in the testa outer cell layers and in specific cell wall thickening. Sclereids are characterized by extensive secondary cell wall formation and are usually non-living at maturity with a callose-rich wall area running parallel to the edge of the seed coat (Bhalla and Slattery, 1984; Serrato Valenti et al., 1993; Ma et al., 2004). In common bean, cell vacuoles of the thick-walled epidermal cells are often completely filled with tannins, indicating that the macrosclereid cells play a key role in hardening of the seed coat (Algan and Büyükkartal, 2000). The contrasting cellular fates above are related to different functional requirements during the course of development: nutrient transport and metabolism to sustain embryo growth during embryogenesis and seed filling and physical and structural requirements as the seed matures to insure protection.

### OMICS ANALYSIS: TOWARD A GLOBAL GENE ACTIVITY PROFILE OF SEED COAT DEVELOPMENT

In general, development of the seed coat has not been characterized at the molecular level to the extent of the embryo and endosperm (Thompson et al., 2009). Transcriptomic and proteomic analyses have been used to dissect the molecular mechanisms underlying the development of the three major seed tissues, including the maternal seed coat in the legume model *Medicago truncatula* (Gallardo et al., 2007; Pang et al., 2007; Verdier et al., 2008) and in soybean (Le et al., 2007; Ranathunge et al., 2010; Miernyk and Johnston, 2013). Seed coat transcriptome and proteome were shown to be highly correlated in *Medicago* and quite distinct from that of the embryo or endosperm (Gallardo et al., 2007). Results highlighted a metabolic interdependence of these three seed components during seed filling, with certain metabolic steps or enzymes being restricted to a particular tissue. An example is several proteases specifically produced in the seed coat that may be important to provide amino acids for protein synthesis within the embryo (Gallardo et al., 2007). Similarly, seed coat specific TFs acting during early seed filling when seed coat and endosperm are active in supplying nutrients to the developing embryo were identified (Verdier et al., 2008) and could represent master regulators of seed coat development and function. More recently, a combined histology and transcriptomic analysis of the *Medicago* seed coat was performed (Verdier et al., 2013a) and a regulatory network-based analysis of transcriptome profiles during *Medicago truncatula* seed maturation was carried out (Verdier et al., 2013b). At 4 to 6 days after pollination (DAP), arrest of cell division occurs, which is compensated by cell elongation in the expanding seed coat. This cell size increase was associated with endopolyploidy and supported by transcriptomics data showing over-expression of “nucleotide metabolism” class genes. In addition, laser capture microdissection and transcriptional profiling were used to identify genes expressed in different sub-regions of soybean seeds (Le et al., 2007; http://seedgenenetwork.net/soybean). Transcriptomic data are not only available for nearly mature seed coat layers (hourglass, palisade, parenchyma) but also for the inner and outer integuments during early seed coat development, providing a source of candidate genes for further functional analyses.

### POLYPHENOLIC COMPOUNDS BIOSYNTHESIS AND ACCUMULATION

**FIGURE 2:**
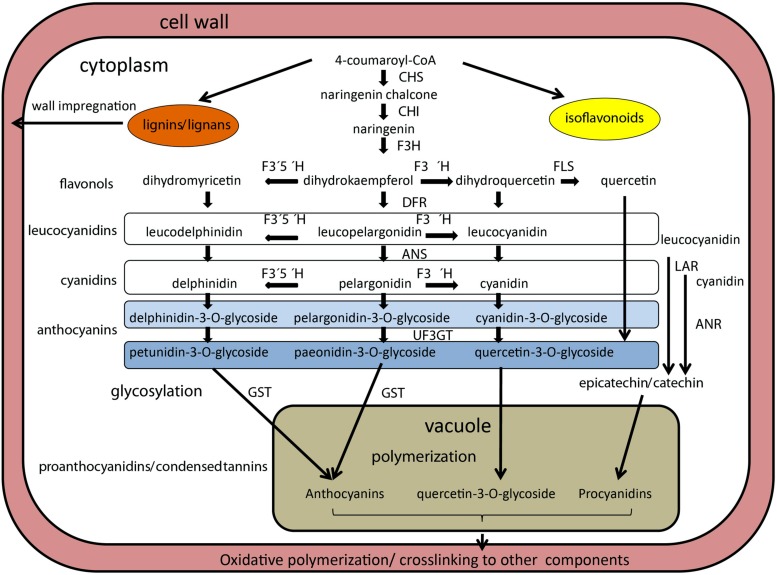
**Schematized representation of biosynthetic pathway of anthocyanins in legume seeds.** Enzymes are indicated in bold italics, metabolic intermediates are in plain text. Enzymes are abbreviated as follows: anthocyanin reductase (ANR), chalcone synthase (CHS), chalcone isomerase (CHI), dihydroxyflavone reductase (DFR), flavanone 3-hydroxylase (F3H), flavonoid 3′5′hydroxylase (F3′5′H), flavonoid 3′hydroxylase (F3′H), glutathione-S-transferasse (GST), leucoanthocyanidin reductase (LAR), UDP-glucose:flavonoid 3-*O*-glycosyltransferase (UF3GT). Leucocyanidins and cyanidins are colorless, while anthocyanins and pro anthocyanins are colored. Based on Zhao et al. (2010), Gillman et al. (2011), Kovinich et al. (2011), and Reinprecht et al. (2013).

The PAs, oligomers of flavan-3-ol units, have received particular attention due to their abundance in seed coats (Dixon et al., 2005; Zhao et al., 2010). PAs are also known as the chemical basis for tannins, polymeric flavonoids that comprise part of the broad and diverse group of phenolic compounds that plants produce as secondary metabolites (Winkel-Shirley, 2001). These are synthesized in the inner integument or endothelium layer. PA biosynthesis and its regulation have been dissected in *Arabidopsis* using *transparent testa* (*tt*) mutants, which regulate production, transport or storage of PAs (Lepiniec et al., 2006), and 20 genes affecting flavonoid metabolism were characterized at the molecular level (reviewed in Bradford and Nonogaki, 2009). Most notably, a set of three types [(TT2, a myeloblastosis family of transcription factors (MYB) protein family member; TT8, a basic-helix-loop-helix (bHLH) proteins; and TTG1, a WD40 protein)] of TFs was characterized that interact to regulate transcription of anthocyanidin reductase (ANR; **Figure 2**), a key enzyme producing the epicatechin building block of PAs (Baudry et al., 2004). Many of these flavonoid biosynthesis pathway genes have been found to affect dormancy of *Arabidopsis* seeds, indicating the role of pigments in this process (Debeaujon et al., 2000). Whether these genes play a similar role in legume seeds remains to be shown. Tannins are also important nutritionally because they can complex with several minerals and proteins in the gastrointestinal lumen, reducing the absorption, digestibility and availability of these nutrients (Brune et al., 1989). In *Medicago truncatula*, seed coat-expressed genes involved in PA biosynthesis and transport (**Figure 2**) have also been identified (Pang et al., 2007; Zhao and Dixon, 2009; Zhao et al., 2010), and two genes implicated in the regulation of PA biosynthesis have been isolated, a WD40-repeat TF (Pang et al., 2009) and a MYB family TF (Verdier et al., 2012).

Legumes vary in the types of PA monomers that are polymerized to form tannins (**Figure 2**). The best studied *Medicago truncatula* PAs are composed primarily of epicatechin and/or catechin units (which yield cyanidin on hydrolysis), with much lower levels of epigallocatechin or gallocatechin (yielding delphinidin) and epiafzelechin or afzelechin (yielding pelargonidin) units. In lentils, there is a balance between catechin and gallocatechin units; and the polymer fraction was more abundant than the monomer and oligomer fraction (Dueñas et al., 2003). In contrast, common beans had mainly catechin monomers (60% on average) in their seed coat tissue, with minor and variable amounts of gallocatechin and afzelechin (Díaz et al., 2010). Other genes affecting PA deposition play roles in the differentiation of the endothelium layer (Dean et al., 2011). This layer is for the most part a single cell layer, but in *Arabidopsis* limited periclinal divisions were observed in the micropylar region (Debeaujon et al., 2003). Interestingly, in *Arabidopsis*, PA accumulation begins in the micropylar region of young seeds approximately at the two-cell stage of embryo development, progressing through the seed body, and ending in the chalazal end at the heart stage of embryo development, a process reminiscent of the asynchronous differentiation of endosperm during seed development. The availability of an endothelium-specific promoter (*BANYULS* gene of *Arabidopsis*) permitted (Debeaujon et al., 2003) testing the effect of the ablation of this cell layer using a promoter:Barnase fusion. The seed, which completely lacked the pigment layer, remained viable, while embryo, and endosperm development were not being obviously affected, but testa-imposed dormancy was reduced. However, other mutants acting earlier (pre-fertilization) on the formation of the integument layers, do affect, or even prevent, seed development (Schneitz et al., 1997; Mizzotti et al., 2012). The importance of pigments for early embryogenesis remains to be tested in legumes, particularly in either *Medicago truncatula* or *Lotus japonicas* models.

The high levels of lignin polymers in the seed coats support the view that they have a primary structural function, i.e., providing mechanical strength and impermeability. Polymerization of soluble phenolics to insoluble polymers has been suggested to be promoted by peroxidases (Gillikin and Graham, 1991) and catechol oxidases (Marbach and Mayer, 1974; Werker et al., 1979), which are abundant in legume seed coats. These individual compounds play important roles in defense against microbial pathogens and other biotic and abiotic stresses, as well as affecting the nutritional quality of products, and because of their health benefits, are of industrial and medicinal interest. Tannins in seed coat are particularly effective against fungal infection (Kantar et al., 1996; Winkel-Shirley, 2001).

### SEED COAT COLOR PATTERN GENES

In common bean, the roles of seed coat color pattern genes such as *Bip* (*bipunctata*) and *Z* (*zonal*) genes in the accumulation of tannin also suggest that tannin first accumulate in the seed coat tissues nearest the hilum (Caldas and Blair, 2009). Patterned seeds require a *t/t* genotype for expression of a partial color, whereas a *T/-* genotype gives seeds that are totally colored. The types (form and extension) of colored patterns are then controlled by the interaction of the *t* gene with the two previously mentioned *Z* gene for patterned color expression on the testa, plus the genes *L* (*limiter*) and *J* (*joker*) which were found to be allelic to each other (Bassett, 1994, 2002, 2007). Finally, the *Z* gene is allelic with the *D* gene, which specifically determines hilum color (Bassett, 1999). Outside the legume family, 10 species were evaluated for condensed tannins in the Brassicaceae, and all of them contained tannins in the hilum including species where no tannins were found in other regions of the seed coat (Marles and Gruber, 2004). The hilum is known to be the point of attachment of the seed to the placental tissue of the ovary, but it also is the place where seed water uptake often begins, a portion of the seed that would need to be protected by tannins which have roles as antifungal metabolites. Notably, white beans, which lack any coloration, usually have no tannins or very low tannin levels and are more susceptible to root rots and other diseases (Ma and Bliss, 1978; Guzmán-Maldonado et al., 1996).

In soybean, six independent loci (*I, T, R, Wp, W1,* and *O*) control the color and distribution of pigments (Bernard and Weiss, 1973; Palmer and Kilen, 1987; Gillman et al., 2011). The best characterized *I* locus (for *inhibitor*, ecoding chalcone synthase) inhibits the production and accumulation of anthocyanins and PAs in the epidermal layer of the seed coat (Bernard and Weiss, 1973). An allelic series for the *I* gene is present in soybean, where the absence of pigmentation is controlled by the dominant allele at the *I* locus, whereas the homozygous recessive *ii* genotype produces a totally pigmented seed coat, and the alternate *i^i^* allele results in pigmentation of the hilum (Tuteja et al., 2004). Most cultivated soybean varieties are homozygous for the *I* gene, resulting in a yellow seed coat. To date, eight chalcone synthase genes have been identified in soybean, all expressed in seed coat (reviewed in Moïse et al., 2005). Chalcone synthase is the first enzyme of the branched pathway of flavonoid biosynthesis (**Figure 2**), and it plays a role in the synthesis of secondary metabolites functioning as UV protectants, phytoalexins, insect repellents, and symbiosis initiators in various plant tissues. Another locus, *T*, affects pubescence and hilum colors, and it induces seed coat cracking. It encodes flavonoid 3′-hydroxylase, which is necessary for the formation of quercetin from kaempferol (**Figure 2**) and is responsible for the hydroxylation of the 3′position of flavonoids, leading to the production of cyanidin pigments (Zabala and Vodkin, 2003). The *W1* locus controls flower color and affects seed color only in an *iRT* background, where *W1* and *w1* alleles give imperfect black and buff seed coat colors, respectively. The *W1* allele encodes flavonoid 3′,5′-hydroxylase (Zabala and Vodkin, 2007) and causes purple flower. The *Wp* locus was suggested to code for the flavanone 3-hydroxylase (**Figure 2**) based on microarray analysis (Zabala and Vodkin, 2005), where the recessive *wp* allele resulted in change from black (*iRTWp*) to light grayish (*irtwp*) color. The *O* locus affects the color of brown seed coat (*irTO*) and has been suggested to code for ANR (Yang et al., 2010). Finally, the *R* locus controls the presence (*R*) or absence (*r*) of anthocyanins in black (*iRT*) or brown (*irT*) seed coat, respectively (Nagai, 1921). The up-regulation of anthocyanidin synthase genes suggests that the *R* locus codes for a regulatory factor (Kovinich et al., 2011). Comparably less is known about pigmentation in pea, especially in relation to the testa. Mendel’s *A* gene, conferring pea flower color and testa pigmentation, has been recently identified as a bHLH TF, exerting thus pleiotropic effects on flower, leaf axils and testa pigmentation (Hellens et al., 2010). However, these traits can be genetically uncoupled, e.g., pea with colored flowers can have nearly non-pigmented seed coat (Smýkal, unpublished). The *b* gene of pea was shown to encode a defective flavonoid 3′,5′-hydroxylase, conferring pink flower color (Moreau et al., 2012). However, none of these two mutations result in alteration of seed dormancy, since they have been identified in cultivated pea lines. Also in lupin, besides the hard-seeded gene *Mollis*, the blue flower and dark seed color gene *Leucospermus* (Clements et al., 2005) confers domestication trait. Although truly wild *Vicia faba* has not identified, a locus was found to be involved in seed dormancy (i.e., *doz*) linked to a locus controlling anthocyanin and pro-anthocyanidin synthesis (i.e., *sp-v*; Ramsay, 1997). Finally, in *Trifolium subterraneum* seeds, a relationship between color, phenolic content and seed coat impermeability was found (Slattery et al., 1982).

### OTHER SEED COAT COMPOUNDS INVOLVED IN PROTECTION

Besides pigments, several active enzymes have been isolated from the legume seed coat. Chitinases were isolated from soybean and are expressed late during seed development (Gijzen et al., 2001). They have been suggested to play a role in plant defense against fungal pathogens (Schlumbaum et al., 1986). Peroxidase is a major component of the protein fraction in mature soybean seed coat where it accumulates in the hourglass cells of the epidermis. Initially involved in the synthesis or modifications of extracellular polymers such as lignin or suberin, peroxidase is believed to have a function in defense when released from the hourglass cells during seed imbibition (Moïse et al., 2005). Furthermore, polysaccharides, such as galactorhamnans, are present in the innermost layer of jack bean (*Canavalia ensiformis*) seeds, and are effective against seed beetles (Oliveira et al., 2001). A well-documented barrier for preventing water entry via the seed coat is suberin accumulation (Nawrath, 2002). Suberin is composed of two distinct types of insoluble polyesters of fatty acid and glycerol. Suberin has been found in the pea testa (Spurny, 1964). Genetic evidence shows that suberin deposition controls seed permeability (Beisson et al., 2007), and glycerol-3-phosphate acyltransferase (GPAT) gene candidates for a role in suberin biosynthesis have been identified in soybean (Ranathunge et al., 2010), *Medicago truncatula* (Verdier et al., 2013a), *Arabidopsis*, and *Melilotus* (Liang et al., 2006; Beisson et al., 2007).

### ROLE OF SEED COAT IN SEED DEVELOPMENT

Thorne and Rainbird (1983) developed a method, adopted by several groups, for measuring phloem unloading from seed coats by excising the immature embryo and recovering assimilates unloaded into the embryo sac. Legume seed coats were thus shown to play a critical role in the lateral transfer of assimilates and other nutrients, prior to their release to the developing embryo (Lush and Evans, 1980; Oﬄer and Patrick, 1984, 1993). The seed coat supplies the zygote with water and oxygen, minerals, certain phytohormones including ABA and IAA, and C and N assimilates in the form of sucrose and amino acids unloaded from the phloem terminals. Further, the legume seed coat is the site of inter-conversions of the principal amino acids unloaded from the phloem, asparagine and aspartate, via asparaginase and aminotransferases, to a composition better adapted for storage protein accumulation, before unloading into t the embryo sac (Murray and Kennedy, 1980; Lanfermeijer et al., 1992). Similarly, sucrose is partly hydrolysed by extracellular invertases prior to entry into the embryo sac, contributing to the maternal control of legume seed development (reviewed in Weber et al., 2005). Cell wall invertases promote assimilate unloading by increasing the sucrose concentration gradient in the unloading zones of the legume seed coat. The glucose released promotes embryo cell divisions, determinant of final seed size (Weber et al., 1997). Solute transfer is also facilitated by the differentiation of specialized transfer cells located in the coat parenchyma or cotyledon epidermis, i.e., at the interface between the seed coat and the embryo (Thompson et al., 2001), as found in the epidermis of faba bean and pea embryos (Bonnemain et al., 1991). Transfer cell development is accompanied by the increased expression of a sucrose-H^+^ transporter gene. Both cell differentiation and gene expression have been suggested to be induced by signals coming from the maternal seed coat or to be elicited by tissue contact. Auxin, ethylene and reactive oxygen species (ROS) have been proposed as inductive signals for transfer cell differentiation (reviewed in Andriunas et al., 2013).

In addition to the production of PAs and other defense-related compounds, the seed coat is an important source of phytohormones for the developing seed, either synthesized *in situ*, or transported from the mother plant. In pea, Nadeau et al. (2011) analyzed GA content and transcript abundances of major enzymes of the GA biosynthesis pathway and studied the consequences of their inactivation in the three seed tissues during development. The results are consistent with a key role for GA in orchestrating seed coat differentiation via the production and turnover active GA forms in the seed coat during early and mid-phase seed development. In legume seeds, cytokinin (CK) concentrations are low (Slater et al., 2013), with a maximum during embryogenesis and at the beginning of the filling stage. Highest CK levels within the seed are found in the seed coat and in the liquid endosperm (Emery et al., 2000). Calculations of CK delivery rates from transport fluids to seeds suggest that CK is synthesized in situ in the seed coat and/or endosperm (Emery et al., 2000). CK was proposed to promote cell division and thus enhance sink strength, increasing solute unloading from the seed coat (Quesnelle and Emery, 2007). Auxin and its conjugated form IAA-Asp play essential roles in controlling pattern formation in the developing legume embryo, and were shown to be the major hormones present in the embryo during the early stage of development (Slater et al., 2013). In the Fabeae tribe of legumes, 4-chloro auxin is the predominant auxin species (Reinecke, 1999). Seed-produced auxin is also transported to the pericarp, where it coordinates pod development with that of the seeds (Ozga et al., 2009; Park et al., 2010).

Abscisic acid accumulates within legume seed (Liu et al., 2010; Slater et al., 2013), and genes involved in ABA biosynthesis were found to be expressed during seed filling in *Medicago truncatula* seed coat, suggesting it could be a source of the hormone (Verdier et al., 2013a). ABA is required for the seed maturation program, acting via the “master regulator” TFs to control storage and late embryogenesis accumulating (LEA) protein deposition and the acquisition of desiccation tolerance (Verdier et al., 2013b). Seed coat-mediated dormancy in *Medicago truncatula* requires ABA (Bolingue et al., 2010), probably produced by maternal seed tissues, as shown in tobacco by the reciprocal crosses of *aba* mutants, defective in ABA synthesis (Karssen et al., 1983; Frey et al., 2004). Developmental signals often result from interactions between two or more hormones. Hence fertilization-stimulated auxin production modulates synthesis of active GA (Dorcey et al., 2009), and the ratio of ABA:GA modulates seed filling (Liu et al., 2010). Liu et al. (2012) found a positive correlation between ABA and IAA concentrations and seed filling rate when comparing varieties of contrasting seed size.

## ROLE OF SEED COAT IN IMPERMEABILITY TO WATER

### STRUCTURAL ASPECTS

In soybean, an anatomical study showed that the only features consistently correlating with seed coat permeability to water were small cuticular cracks (Ma et al., 2004). These cracks were present in soft but not hard seeds (within the studied cultivated genotypes). The initial penetration of water into soft seeds typically occurred on the dorsal side, the location of the majority of the cracks (Ma et al., 2004). Besides anatomical differences, chemical analysis resulted in the identification of a seed coat cutin with unusual chemical composition, lacking typical mid-chain hydroxylated fatty acids but being relatively rich in other types of hydroxylated fatty acids (Shao et al., 2007). The cuticle of the impermeable soybean cultivar (again, no wild soybean was studied) contained a disproportionately high amount of hydroxylated fatty acids relative to that of the permeable ones. According to the results of Shao et al. (2007) and those of Ma et al. (2004), the difference between hard and soft soybean seeds was based on the composition and continuity of the outermost seed cuticle and the presence of small cracks in the cuticles of the latter. The differences in chemical composition were rather subtle, and the authors speculated that crucial elasticity of soybean cuticle might be related to its association with carbohydrates (Ranathunge et al., 2010).

The site of water entry into the seed coat is still a matter of debate (Meyer et al., 2007; Ranathunge et al., 2010). Various explanations were proposed to account for the different permeability of the seed coats to water in hard and soft seeds including tightly bound palisade cells (Corner, 1951; Ballard, 1973), thickened seed coat tissues (Wyatt, 1977; Miao et al., 2001), lack of pits (Chachalis and Smith, 2001), presence of endocarp deposits (Yaklich et al., 1986), dark color (Wyatt, 1977), closed hilum and/or micropyle (Hyde, 1954; Ballard, 1973; Rolston, 1978; Hu et al., 2009), modifications of the outer tangential walls of palisade cells (Werker et al., 1979), a prominent light line of the palisade cells (Harris, 1984, 1987), presence of water gaps in the lens (Dell, 1980; Hanna, 1984; Serrato Valenti et al., 1986, 1989, 1993; Serrato-Valenti et al., 1995; Van Staden et al., 1989; Shen-Miller et al., 1995; Morrison et al., 1998; Baskin et al., 2000; Burrows et al., 2009; Hu et al., 2009), as well as cracks in the cuticle of the seed coat (Morrison et al., 1998; Hu et al., 2009). In some legume species, such as *Vigna*, *Robinia, Acacia*, and many tropical and sub-tropical legume species (Karaki et al., 2012), water enters the seed a specialized structures such as the hilar slit and water gap (e.g., strophiole or lens; Gopinathan and Babu, 1985; Kikuchi et al., 2006) opens. The morphological characteristics of these water gaps can vary between species. It is thought that water gap structures act as an environmental sensor to fine-tune germination to coincide with an environment that provides the best chance for seedling survival and ecosystem colonization. Water gaps are usually associated with areas of the seed coat where natural openings in the ovule occurred during the seed development such as the hilum, micropyle, and chalaza. Although the soybean seed coat composition and structure are modified at the hilum and micropyle areas, these are thought not to contribute to the initial uptake of water (Ma et al., 2004). Instead, in soybeans, water mainly enters through small cracks in the seed coat to reach the embryo (Chachalis and Smith, 2000). There is some controversy on this issue, since other studies have suggested water uptake through the hilum (McDonald et al., 1988). In a related Phaseoleae tribe, the hilum, micropyle and lens have been proposed to be water entrance points for example, in *Phaseolus lunatus* (Korban et al., 1981) and *Phaseolus vulgaris* (Agbo et al., 1987).

In subfamilies Caesalpinioideae and Mimosoideae, cracks develop in the extrahilar region or in the hilum that allow water to enter the seed (Gunn, 1984, 1991; Hu et al., 2009). The hilum of faboid seed, except in flattened seeds, has a separation in palisade cells called the hilar groove (Lhotská and Chrtková, 1978), which is a diagnostic botanical feature. Hyde (1954) reported that this groove acts as hygroscopic valve, that prevents water entry from the outside but permits water to leave seed interior during maturation and drying. The seed coat becomes impermeable as drying occurs. Since the seed remains dry until released from dormancy, it has the potential to remain viable for many years. Seed dormancy not only prevents immediate germination, but it also regulates the time, conditions, and location where germination will occur.

The occurrence of precocious germination under humid conditions indicates that seeds can become germinable prior to maturation drying. There are contrasting results concerning whether some degree of drying is required for switching seeds from dormant to a germinative mode. Prior to desiccation, the seed undergoes developmental changes and mostly anabolic metabolism associated with formation of the embryo and its surrounding structures and the deposition of the major storage reserves. Following desiccation and rehydratation, seed metabolism becomes largely catabolic to support germination. Thus, for most desiccation-tolerant (orthodox) seeds, including legumes, maturation drying clearly switches the seed to a germination mode upon subsequent rehydration. Desiccation-intolerant (recalcitrant) seeds can effect this switch without dehydratation, as can orthodox seeds developing inside fleshy fruits (tomatoes, melons), having the capacity to germinate without maturation drying. However, premature removal of the seeds from the fruit inevitably severs the maternal connections. In the case of common bean and soybean seeds, it was reported that fresh developing seeds are unable to germinate without prior dehydratation. On the other hand, it was found that *Phaseolus* seeds near the end of the seed filling period are capable of germinating within the fruit with their funicular connection intact if water was injected into the pods. To survive in dry state (with less than 10% moisture content on a dry-weight basis), a seed has to avoid damage to its cellular components, both during water loss and upon subsequent rehydratation. Damage does occur, but is limited to a level that can be repaired and that involves accumulation of non-reducing sugars (sucrose, trehalose), oligosaccharides (raffinose) and other solutes such as proline, glycinebetaine (cited in Bewley et al., 2013). In addition to these metabolites, large number of LEA proteins, including dehydrins and heat shock proteins (HSPs), are expressed, both responding positively to ABA.

### SEED COAT PIGMENTATION AND DORMANCY

Seed coat pigmentation was shown to correlate with imbibition ability in several legumes. Browning of seed coat during maturation was found to be associated with its impermeabilization in common bean (Caldas and Blair, 2009; Díaz et al., 2010), chickpea (Legesse and Powell, 1996), yardlong bean (Kongjaimun et al., 2012), soybean (Liu et al., 2007), faba bean (Ramsay, 1997), and pea (Marbach and Mayer, 1974; Werker et al., 1979).

The genes or quantitative trait loci (QTLs) for seed color and loss of seed dormancy in azuki bean (*Vigna angularis*) were shown to be closely linked, and there is a significant correlation between these two traits (Isemura et al., 2007). A positive correlation has also been found between phenolic content, the activity of catechol oxidase and seed dormancy in wild pea seeds (Werker et al., 1979). Recently, epicatechin, cyanidin 3-*O*-glucoside, and delphinidin 3-*O*-glucoside were specifically isolated in wild but not in cultivated soybean seed coats, with epicatechin showing a significant positive correlation with hardseededness (Zhou et al., 2010). Combined analysis of seed coats of black vs. brown isogenic lines of soybean, indicated over-accumulation of anthocyanins, altered procyanidin, and reduced flavonol, benzoic acid, and isoflavone content in black seeds, as a result of altered transcription of numerous biosynthetic pathway genes (Kovinich et al., 2011). Increased β-1,3-glucans (callose) deposition in cell walls during maturation is associated with increased dormancy in a number of species (Finch-Savage and Leubner-Metzger, 2006), while β-1,3-glucanases, which break down callose are associated with dormancy release. Other soluble phenolic compounds, such as coumarin and chlorogenic acid, and their derivatives, or ferulic, caffeic, sinapic acids occur in the coats of many seeds. These may inhibit seed germination and could be leached out into the soil where they may inhibit neighboring seeds (a form of allelopathy). Phenolic compounds of legume seeds, however, participate in nodulation by acting as chemoattractants, promoting rhizobial growth, and inducing transcription of nodulation genes in symbiotic bacteria (Mandal et al., 2010).

### GENETICS OF LEGUME SEED DORMANCY

Seed dormancy is a monogenic trait in lentil (Ladizinsky, 1985), narrowleaf lupin (Forbes and Well, 1968), yardlong (Kongjaimun et al., 2012), rice bean (Isemura et al., 2010), mungbean (Isemura et al., 2012), associated with one to two loci in common bean (Koinange et al., 1996), and two to three loci in pea (Weeden, 2007). In this last case, control of seed dormancy was via testa thickness, testa impregnation and structure of the testa surface. In azuki bean (Isemura et al., 2007; Kaga et al., 2008), four to six QTLs were associated with field germination, time of germination, testa permeability, winter survival of seeds in the soil, days to germination of winter-surviving seeds in the field and water content in seeds. In recently (∼100 years) domesticated lupin, *Lupinus angustifolius*, the hard-seeded gene *mollis*, and the blue flower and dark seed color gene *leucospermus* (Clements et al., 2005) are two of the key domestication traits. A molecular marker linked to the recessive *mollis* gene was discovered and applied to lupin breeding (Boersma et al., 2007; Li et al., 2012), where wild material was used to provide new diversity. Interestingly, Ladizinsky (1985) found that two different monogenic systems operated in crosses of *Lens orientalis* and *Lens ervoides*, with cultivated lens, *Lens culinaris*. In the former, the allele for dormancy was dominant, but in the latter it was recessive. The gene for dormancy in *Lens orientalis* appeared to be linked to another one controlling pod shattering. Similarly, interspecific crosses between *Vicia sativa,* and a closely related species, *Vicia cordata* suggested a two-gene system (Donnelly et al., 1972). Breeding has allowed the development of soft-seeded summer and hard-seeded winter lines of *Vicia sativa* (cited in Büyükkartal et al., 2013). As mentioned earlier, the testa is of maternal origin. The maternal control is clearly demonstrated when a soft-seeded (e.g., with no or low testa imposed seed dormancy) plant is used as a female parent and crossed with a hard-seeded wild type. The F_1_ seed is soft-seeded, and all the resultant F_2_ seeds are hard-seeded, including those individual seeds possessing the homozygous soft-seediness genotype, as shown in lupin (Li et al., 2012).

## DORMANCY-BREAKING REQUIREMENTS OF LEGUME SEEDS

Many legume seeds are known to be long-lived, and they are frequently found in seed bank surveys. *Melilotus* seeds survived for 17 years in the soil (Bibbey, 1948), seeds of *Malva rotundifolia* (Malvaceae) lived for 120 years in burial seed experiment (Telewski and Zeevaart, 2002) and seeds of *Astragalus distortus* germinated 24 years after sowing under near-natural conditions (Baskin and Baskin, 2014). The breaking of dormancy under natural conditions is only partly understood. As mentioned above, water and temperature are two principal environmental regulators of seed germination. In many ecosystems, fire is also an important factor and there is no doubt that germination is promoted by fire-induced heat treatment in species such as *Acacia sp*. in the legumes family (Sabiiti and Wein, 1987; Hanley and Fenner, 1998). It is not understood, however, how germination is regulated in ecosystems with a temperate climate, where fire occurs very rarely and where fluctuations in daily soil temperature are rather limited to a maximum of 10–15°C. Since temperature is relatively constant in its seasonal variations, it is arguably the most important environmental factor to synchronize seed germination with conditions suitable for seedling establishment. This is certainly valid for seasonal climate types, but in arid and semi-arid regions water may be the most important factor, whereas in the humid tropics variations in temperature and water availability appear to be virtually absent. For temperate regions species, germination in summer is accompanied by a higher risk of seedling loss due to drought or shading by leaf canopies, whereas in autumn the seedlings experience a reduced length of the growing period and risk frost damage (Probert, 2000). It has been proposed that hard seeds become permeable to water after mechanical abrasion by soil particles, decomposition of the seed coat by microbial action, ingestion and passage through the digestive tracts of an animal or by cracks in the coat caused by partial seed consumption, but little evidence is available to support these views (Gogue and Emino, 1979; Baskin and Baskin, 2000; Dueberrn de Sousa and Marcos-Filho, 2001; Fenner and Thompson, 2005). Hardseededness has been shown to protect seeds during the passage through the digestive tract (Simao Neto et al., 1987), to extend seed longevity (Mohamed-Yasseen et al., 1994) and persistence in soil seed banks (Shen-Miller et al., 1995). Dalling et al. (2011) postulated that species with physical seed dormancy rely on physical defenses to exclude predators and pathogens. In this case, rapid seed germination cannot be used to escape pathogens at the emergence stage. Recently, it was proposed that hard seeds are an anti-predator trait that evolved in response to selection by small mammal seed predators (Paulsen et al., 2013). Seeds of two legume species with dimorphic seeds (“hard” and “soft”), *Robinia pseudoacacia* and *Vicia sativa*, were offered to desert hamsters. Volatile compounds released from imbibed seeds attracted the hamsters to the seeds, but the animals could not detect buried hard or dry soft seeds. Correlations between the dormancy release mechanism and ecological habitat were tested in four legume species (van Klinken and Goulier, 2013): two wetland species (*Mimosa pigra* and *Parkinsonia aculeata*), both dispersed primarily by water and two terrestrial species (*Acacia nilotica* and *Prosopis pallida*), both dispersed primarily through vertebrate herbivores. Seed viability was largely unaffected by temperature or moisture regime, although it differed with species and was lower for non-dormant seeds.

There are two steps in breaking physical dormancy with high temperatures. At first, the preconditioning phase will occur if seeds are at constant temperatures, and the rate at which this stage is completed increases with an increase in temperature. Seeds prevented from drying (by blocking the hilum) during the first stage are more likely to become water-permeable in the second stage than those that dehydrate further during stage one. The second stage (when seeds become permeable) requires fluctuating temperatures for maximum loss of dormancy (Baskin and Baskin, 2014). Taylor (1981) suggested that thermal degradation occurs during the first stage, which results in weakening of the lens. In the second stage, physical expansion and contraction associated with temperature fluctuations cause cells in the lens to open. There are also two steps in breaking dormancy by low winter temperatures, as found in temperate-zone species. Study of *Melilotus alba*, *Vicia villosa*, and *T. pratense* showed that during a first step, low winter temperatures make seeds sensitive to alternating temperatures, and during a second step, these alternating regimes occurring in early spring cause the sensitive seeds to become water-permeable. Further, cycles of germination (but not dormancy break) occur because spring temperatures might not be adequate to open the water gap of sensitive seeds. Thus, seeds may lose their sensitivity and have to go through another winter to become sensitive again. Seeds can only respond to alternating spring temperatures if they become sensitive during the winter (Baskin and Baskin, 2014).

The alternating temperatures that break the physical dormancy of a seed depend on the amplitude of the fluctuation. Clover (*T. subterraneum*) seeds become sensitive in response to temperatures that fluctuate between 30 and 60°C over a period of several weeks or months, the fluctuations occuring on open soils in Mediterranean-line climates, and subsequently their seed coats become permeable for water (Quinlivan, 1971; Taylor, 1981, 2005; Moreno-Casasola et al., 1994; Taylor and Ewing, 1996; Taylor and Revell, 1999, 2002; de Souza et al., 2012). The proportion of soft/hard seeds seems to vary from year to year (Roberts and Boddrell, 1985), presumably as a result of climatic effects. The influence of seasonal factors on the germination of impermeable seeds of 14 herbaceous legume species was studied by Van Assche et al. (2003). Six species (*Medicago lupulina, Melilotus, Lotus, T. pratense, T. repens*, and *Vicia cracca*) showed a marked seasonal cycle with high germination rates in spring. *Medicago arabica, T. dubium*, and *Vicia sativa*, which are typical winter annuals, germinated mostly in summer and autumn, while *Lathyrus aphaca, Lathyrus nissolia*, and *Vicia hirsuta*, germinated in all seasons except summer (Van Assche et al., 2003). The importance of daily temperature fluctuations in breaking physical dormancy was shown for several legume species. The percentages of impermeable seeds of *Stylosanthes humilis* and *Stylosanthes hamata* begun to decline in northern Australian pastures in September (early spring) when mean monthly maximum and minimum temperatures were about 67 and 28°C, respectively. The number of dormant seeds decreased until December (early summer) when rains stimulate all permeable seeds to germinate. Dormancy was not broken from January to August, when daily maximum and minimum temperatures were less than 55 and 25°C, respectively (McKeon and Mott, 1982). Impermeable seeds of *Indigofera glandulosa* become permeable (Bhat, 1968) by exposure to hight temperatures (up to 60°C). Impermeable seeds of *Lupinus digitanus, Lupinus luteus, Medicago trilobus*, and *T. subterraneum* subjected to alternating temperature regimes had highest germination rates under temperature regimes of 60/15°C for 4 months (Quinlivan, 1961). Dormancy loss was determined by the maximum daily temperature, provided there was a minimum daily temperature fluctuation of at least 15°C. The maximum daily temperatures required for loss of dormancy varies with the species: *T. subterraneum*, 30°C; *T. hirtum, T. cherleri, T. cernum,* 40°C; *Medicago truncatula, Medicago littoralis, Medicago polymorpha,* 50°C; and *Lupinus varius*, 60°C (Quinlivan, 1968). Such temperature fluctuations are prevented by seeds shaded by plant litter or by soil burial. Continuous moisture of substrate could be an important clue for some seeds to become water permeable. However, this factor has received comparably less attention in the literature. Several species did not imbibe seeds for long periods, up to 3 years for *Stylobasium spathulatum* among Surianaceae (Baskin et al., 2006). The seeds of *Acacia nilotica*, which grows along rivers in Sudan and Egypt and is subjected to flooding, imbibe and germinate at best when soaked for 18 weeks (prolonged submersion decreases germination rates again), which corresponds to the average duration of annual flooding periods (Warrag and Eltigani, 2005). There is also a promoting effect of seed burial in soil which could be related to increased moisture or microbial action (Baskin and Baskin, 2000). Testing of seed germination in presence or absence (using sterilized seeds) of microbes resulted in increase of germination for the former. This was most pronounced in *Vigna minima* seeds, which imbibed within 186 h in the presence of soil suspension but failed to do so in pure water for 30 days (Gopinathan and Babu, 1985). Similarly, passage through the animal’s digestive tract, often results in better germination of impermeable seeds. It is assumed that it acts via acid enzymatic digestion of testa or mechanical scarification (cited in Baskin and Baskin, 2014). Interesting interaction was found between the *Acacia sp*. seeds, bruchid beetles that lay eggs on the seeds and mammals and birds that feed on the seeds. Gazelles feed on pods of *Acacia sp*. in the Negev Desert of Israel and disperse the seeds, which have superior germination as a result. Moreover, the seeds infected with bruchid larvae germinate with higher rates (Halevy, 1974). Seeds containing a bruchid beetle larvae are more likely to germinate before the parasite destroys the embryo if seeds go through the digestive tract of an animal (Pellew and Southgate, 1984). On the other hand, these insect larvae cause substantial proportion of the otherwise water-impermeable seeds to imbibe and germinate, despite part of the seeds have destroyed embryo as shown for *Acacia sp., Vicia sativa, Ulex europaeus*, and *Gleditsia japonica* (cited in Baskin and Baskin, 2014).

Some species produce heterogeneously colored seeds with different degrees of hardness. For example, the tree legume species *Senna obtusifolia* produces 90% hard-coat seeds with 10% soft-coat seeds (in Baskin and Baskin, 1998). 82–93% of the soft-coat seeds germinated, while only 15–32% of the hard-seed-coat ones germinated. This heterogeneity may be an important eco-physiological strategy, since soft-coat seeds can germinate in the spring in temperate regions, whereas hard-coat seeds cannot germinate until late spring–summer, when high temperatures cause an increase in seed-coat permeability. The seeds of the legumes *Adenocarpus decorticans, Astragalus granatensis ssp. Granatensis*, and *Cytisus reverchonii* (all endemic to the Betic Cordillera, Spain) collected at different altitudes, required different temperature for germination (Angosto and Matilla, 1993).

Several artificial techniques are used to break physical dormancy in seeds, including mechanical, thermal and chemical scarification, enzymes, dry storage, percussion, low temperatures, radiation and high atmospheric pressures (Baskin and Baskin, 2014).

## LEGUME SEED DORMANCY AND DOMESTICATION

**FIGURE 3:**
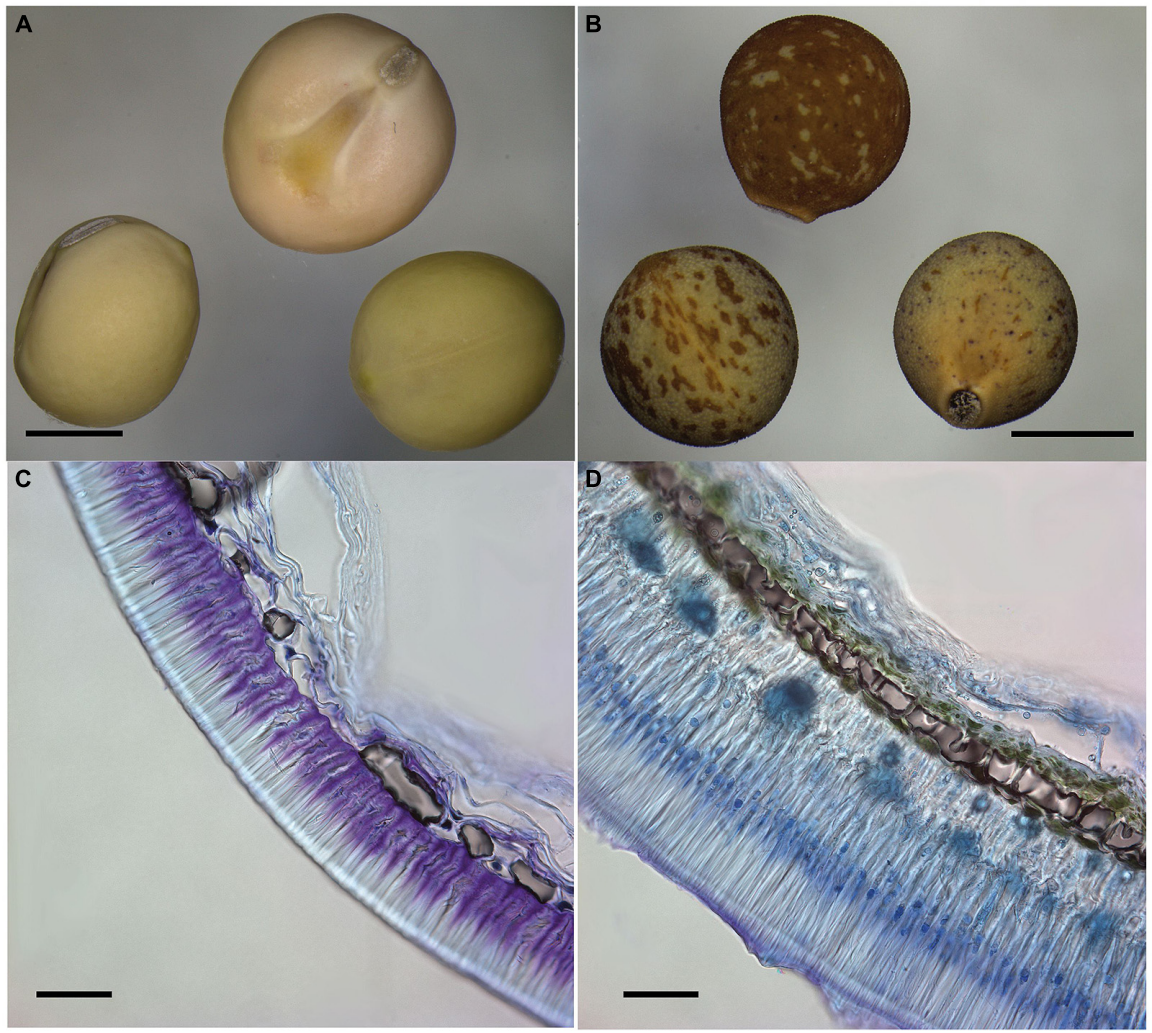
**Comparison of cultivated and wild pea seeds.** Macrograph of modern cultivated pea cv. Cameor with transparent testa **(A)** and wild *Pisum sativum* subsp. *elatius* JI64 with pigmented testa and visible rough (gritty) testa surface **(B)**. Scale bars = 3 mm. Transversal section of toluidine blue-stained seed coat of domesticated *Pisum sativum* cv. Cameor **(C)** and wild *Pisum sativum* subsp. *elatius* JI64 **(D)**. Scale bars = 50 mm.

The development of agriculture was one of the key transitions in human history, and a central part of this was the evolution of new plant forms that were selected and became domesticated crops. The domestication of wild plants into crop plants can be viewed as an accelerated evolution, the result of both human and natural selection (Abbo et al., 2012, 2014). Domestication is often described as a quality in which morphological (and genetic) changes are found amongst cultivated plants in comparison to those in wild populations (Hancock, 2012; Zohary et al., 2012). These domestication-triggered changes represent adaptations to cultivation and human harvesting, accompanied by genetic changes. A common set of traits has been recorded for domesticated, but otherwise un-related crops, which collectively have been called the “domestication syndrome” (Harlan, 1971; Hammer, 1984). These traits are linked to successful early growth of planted seeds and include loss of germination inhibition and increase of seed size (**Figure 3**). Members of the Fabaceae have been domesticated in parallel with cereal domestications (Abbo et al., 2009). One of the major differences between the wild progenitors of Near Eastern grain legumes and cereals concerns the low germination rate imposed by the hard seed coat of these legumes (Ladizinsky, 1979, 1985; Werker et al., 1979; Abbo et al., 2009, 2014; Fuller and Allaby, 2009). Timing of seed germination is thus one of the key steps both in natural and agricultural ecosystems and is a major factor for crop production. In contrast to wild species, crops tend to germinate as soon as they are imbibed and planted making seed dormancy a potentially unwanted trait. The selection acts on loss of dormancy during cultivation (Fuller and Allaby, 2009). Seed imbibition also has a crucial role in the ability of most grain legumes to undergo cooking. Hence, reduction of seed coat thickness has led to a concurrent reduction of seed coat impermeability. Ladizinsky (1987) argued that the very low germination rates in wild pulses, in particular lentil, would have precluded their successful cultivation on the basis of very low yields from planted seeds. He therefore suggested that hunter–gatherers must have selected wild mutants with quick germination for cultivation (Ladizinsky, 1987; Weiss et al., 2006). That germplasm could have been part of a “pre-cultivation domestication” process. The experimental harvest of wild lentils by Abbo et al. (2008) provided strong support for Ladizinsky’s (1985, 1987, 1998) arguments. This also holds true for peas, where intact wild seeds which have a germination rate of only 2.6–7% in a given year (Abbo et al., 2011, 2014). These results suggest that free germination was a more important trait for the domestication of wild pea (and possibly lentil and chickpea as well) than the mode of seed dispersal. However, too low seed dormancy levels reduce seed quality and may trigger pre-harvest sprouting, which can occur in cereals. Therefore, seeds of crop plants require a well-balanced level of seed dormancy. Pre-harvest sprouting is rarely a problem in legume seeds since they are produced within a pod. Similarly, high-quality mungbean and common bean seeds are difficult to produce in humid tropical regions because of susceptibility to weather damage, which, however, can be mitigated by hardseededness (Hamphry et al., 2005). Whilst seed dormancy has been largely removed in grain legume crops, it remains in a number of important fodder crops and less domesticated species. Moreover, even in highly domesticated legume crops such as soybean, which has been selected for seeds that imbibe water rapidly and uniformly, some varieties produce some hard seeds (Rolston, 1978). This constitutes a major problem during food processing where rapid and uniform hydration is important for the production of quality foods.

## CONCLUSION

The seed testa plays important roles in seed development and the beginning of a new plant generation. The seed coat provides not just structural and protective functions, but as discussed in this review, has a decisive role in timing of seed germination of legumes by regulating water uptake. This control is fundamental under variable natural conditions where the establishment of young plants might influence the species evolutionary success. Such control was largely removed from domesticated crop plants, which are largely characterized by immediate seed germination. Moreover, water uptake allows us as well as early farmers, to cook and make legume seeds edible, thus providing important protein levels to the human diet. Although we have a good understanding of the genetic control of germination, we are just starting to identify the genes involved in this process. Although not formally demonstrated, the main testa pigments, PAs, are hypothesized to play a role in seed coat permeability. These pigments are also known as antioxidants with beneficial effects on human health, including cardioprotective, anticancer, and anti-inflammatory roles. These compounds also protect animals by binding to proteins in feed and slowing fermentation in the rumen, thereby reducing microbial production of methane.

Currently available analytical tools applied to a widening range of germplasm will help dissect germination in legumes more precisely. Detailed structural and chemical description of crop seed coats will provide the comparative basis for further studies and might lead to the identification of novel phenolic substances with potential health and nutritional benefits.

## Conflict of Interest Statement

The authors declare that the research was conducted in the absence of any commercial or financial relationships that could be construed as a potential conflict of interest.
